# Nonmetastatic Ewing's Sarcoma of the Lumbar Spine in an Adult Patient

**DOI:** 10.1155/2012/165289

**Published:** 2012-10-24

**Authors:** Maurizio Iacoangeli, Mauro Dobran, Alessandro Di Rienzo, Lucia Giovanna Maria di Somma, Lorenzo Alvaro, Elisa Moriconi, Niccolò Nocchi, Maurizio Gladi, Massimo Scerrati

**Affiliations:** Dipartimento di Neurochirurgia, Ospedali Riuniti di Ancona, Università Politecnica delle Marche, Via Conca No. 71, 60020 Ancona, Italy

## Abstract

Although the spine is frequently involved in metastatic Ewing's sarcoma, primary involvement of the spine, beside sacrum, is much less frequent, especially in adult patients. Because of the low incidence of these tumors, there are currently no clinical guidelines outlining their management and a multitude of therapeutic strategies have been employed with varying success. The definitive management of Ewing's sarcoma of the spine, as in other locations, could include the combination of three main modalities: aggressive surgery, radiotherapy, and combined chemotherapy. Whenever possible, en bloc spondylectomy or extralesional resection is preferable, providing a better oncological result with a longer survival and a better preservation of the spine biomechanics. This is the lesson we learned about the case, we present here, of nonmetastatic lumbar localization by Ewing's sarcoma in as adult patient.

## 1. Introduction

Since its first description by James Ewing in 1921, Ewing's sarcoma represented a strange kind of malignancy. Poorly differentiated tumor of uncertain histogenesis with a variable biologic behavior, it presents heterogeneity inside. It is common in Caucasians and rarely arises in individuals of African and Asian ancestry. Currently, we talk about the Ewing family of tumors which comprises Ewing's sarcoma (EWS), extraskeletal EWS, primitive neuroectodermal tumor (PNET) of bone and soft tissue, and chest wall tumor (Askin tumor) [1–4]. The translocation t(11; 22) (q24; q12) is identified in more than 90% of cases, and it is the landmark to differentiate EWS from other small, round blue cell tumors [1, 2]. The most common primary sites of involvement are the pelvis, femur, tibia, and fibula. In the primary vertebral EWS, the division of the spine into nonsacral (cervical, dorsal, and lumbar) and sacral (sacral and coccygeal) is important and is dictated by the different behavior of EWS in these two regions, being sacral EWS more aggressive and less responsive to therapy [1, 4–7]. Spinal involvement by EWS most commonly results from metastasis in advanced stages. Ewing sarcoma originating from the spine is rare, and extremely rare if the sacrum is excluded. Most studies on EWS involving the mobile spine are limited to case reports [1, 6–8]. EWS is one of the most aggressive bone tumors with high proliferative and invasive potential, presenting a confusing variety of imaging manifestations that can mimic other diseases, especially in adult patients who already suffer of other spine pathologies [9–13]. In contrast to long bone involvement, delays in spinal EWS diagnosis may occur because symptoms may not be present until neurological deficits occur. EWS is the second most common primary bone malignancy in childhood and adolescence with peak age from 15 to 17 years and a slight male predominance [14, 15]. The occurrence in adult age is quite rare [1, 7]. Definitive diagnosis requires cytological and immunohistochemical analysis of a pathologic specimen. To date, the number of reported cases of EWS with primary localization on mobile spine in adult patients remains small and they are all sporadic reports. This is the reason why there are still several concerns about the optimal treatment, especially in adults.

## 2. Case Presentation

**Figure 1:**
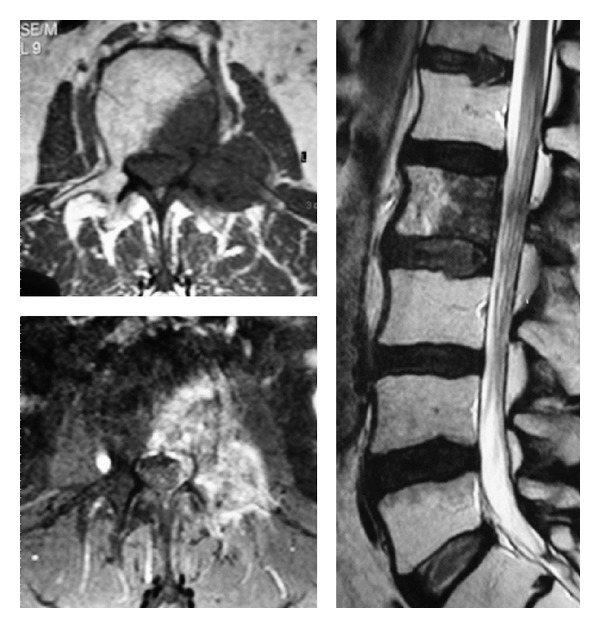
Preoperative MRI images showing an enhancing mass occupying the left hemivertebra, pedicle, and hemilamina of L2.

**Figure 2:**
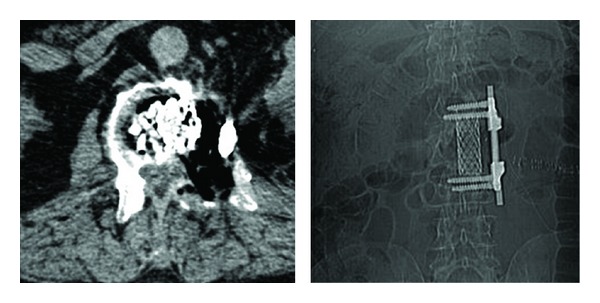
Postoperative CT scan demonstrating the tumor removal and the anterior column reconstruction.

**Figure 3:**
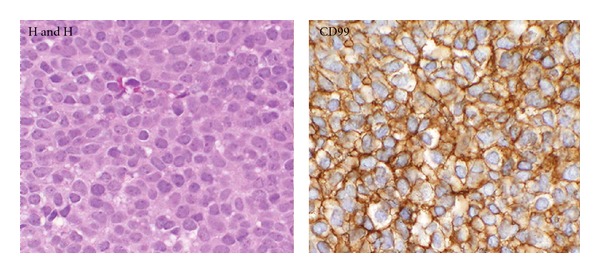
Histopathological images revealing the typical aspect of a densely cellular “blue tumor” composed of small round cells uniformly arranged in sheets and with strong membranous CD99 immunoreactivity (H&H stain ×20, CD99 immunohistochemical stain ×20).

A 58-year-old man with no significant medical history came to our attention after a 3-month history of a progressive worsening, nocturnal, bilateral buttock cramping associated to a left predominant back pain. Paresthesias with tingling were also present on the left leg. No abnormalities were found on neurological examination, beside a diffuse tenderness in the lower back and a restriction of the active flexion-extension motion. The passive straight leg-raising test was positive at more than 45°. He had a low-grade fever (37.4°C). Laboratory tests revealed a mild leukocytosis (8700/L). Erythrocyte sedimentation rate and C-reactive protein level were elevated. All other parameters were normal. Lumbosacral MRI revealed a large, enhancing mass occupying the left hemivertebra, pedicle, and hemilamina of L2 with paravertebral soft tissue extension (Figure 1). The thecal sac was mildly compressed. Staging studies (technetium-99 total body bone scan and total body CT scan) were negative for other neoplastic localizations of the disease. The patient was operated on by a lateral retroperitoneal approach. Intraoperatively, the lesion was highly vascular, brownish, friable, and with a greyish to white cheesy material inside it. Although the tumor was well circumscribed, it did not present a true capsule and it was associate to a complete destruction of the normal vertebral body structure. Resection was up to apparent normal bone, well beyond the limits of the involved vertebral body as disclosed by MRI. Reconstruction was made by interbody titanium mesh and lateral screws/rod expandable fixation (Figure 2). Postoperative course was uneventful. The histopathological examination revealed a typical densely cellular “blue tumor” composed of small round cells uniformly arranged in sheets. Cells had a high nucleocytoplasmic ratio. It was immunonegative for synaptophysin, leucocyte common antigen, neurofilament protein, and neuron-specific enolase but diffusely immunopositive for CD99 (Figure 3). Reverse transcription-polymerase chain reaction analysis confirmed the presence of an EWS-FLI-1 translocation (11; 22). The morphological and immunohistochemical profile was indicative of Ewing's sarcoma. After 3 weeks from surgery, the patient started radiotherapy receiving 6000 cGy to the tumor bed plus 2 cm margin in 30 fractions. Concomitant chemotherapy was also administered. The chemotherapy agents and doses used for the patient included vincristine (maximum dose 1.8 mg), doxorubicin (75 mg/m^2^), and cyclophosphamide (1.2 g/m^2^) alternating with etoposide (100 mg/m^2^/5 days) and ifosfamide (1.8 g/m^2^/day/5 days) every 3 weeks. Five cycles of vincristine, doxorubicin, and cyclophosphamide (VDC) and 4 cycles of vincristine, cyclophosphamide (VC) alternating with ifosfamide, etoposide (IE) (8 cycles), and associated to mesna rescue were administered for a total of 17 cycles. Unfortunately, after 27 months from surgery, the patient was admitted again to our hospital for progressive paraparesis. MRI disclosed a massive intradural dissemination of the lesion with nodules seeding from D8 up to S2. The patient was operated again for decompression at the level of D8 where the mass was more consistent. The histologic examination confirmed the diagnosis of EWS, and the patient died few weeks later.

## 3. Discussion

EWS remains for several aspects an anomalous tumor with bizarre behavior. Hense et al. described an unexplained, intrinsic major aggressive behavior of axial tumors [16]. They postulated that the pelvis possesses an additional effect on tumor aggressiveness. Animals studies confirmed that axial and appendicular EWSs behave differently [1, 2, 16, 17]. These data may collectively suggest biologic differences between axial and appendicular EWSs that ultimately result in prognostic differences. Axial location, large size, presence of metastases at diagnosis, and positive margins all predicted poor overall survival [6, 14, 18, 19]. Tumors belonging to the Ewing family are devastating malignancies that appear to be more common than it has previously been reported [15]. The peak age for EWS is 15 to 17 years, being rarer in adults [1, 7, 14, 15]. The impact of age on the prognosis of EWS remains controversial. In certain series, older age has been associated with a worse clinical outcome [5, 6, 14], yet others have been unable to demonstrate a significant difference based on age alone [4]. In adults, diagnosis is even more difficult, being EWS possibly confused with other more frequent diseases (i.e., degenerative spine). Images are also not specific and can also be misinterpreted. The tumor has been misdiagnosed as pseudohemangioma, neuroblastoma, aneurismal bone cyst, and vertebra plana (Langerhans cell histiocytosis) [7–13]. Because of the low incidence of these tumors in adults patients, the available epidemiology is likely unreliable and there are currently no standard clinical guidelines outlining their management. Because no standard management guidelines exist for treating these tumors, a multitude of therapeutic strategies have been employed with varying success [3, 5, 14, 18–24]. The approach to therapy for pediatric patients is driven by clinical trial-based protocols, whereas adult treatment is often institution-specific [14]. Some adult centers follow pediatric protocols, other centers modify their regimens to better suit this older population. Thus, the ideal treatment strategy for adults with EWS remains undefined. Most of the mortality is due to the metastatic disease, which occurs in approximately 30% of patients [1, 3, 19, 20]. Our patient died for it, despite an aggressive surgical and radiochemotherapeutic treatment. Most patients with Ewing's sarcoma of the spine are firstly treated with systemic chemotherapy [3, 18, 21, 23, 24]. This usual attitude was not possible in our case because the diagnosis was made at operation. During the last two decades, the outcome has improved in patients with localized disease. However, advances in the treatment of Ewing's sarcoma have not impacted on the outcome of patients with large volume and metastatic disease [1, 3, 6, 14, 18, 22]. Approximately 25% of patients present with metastatic disease at diagnosis. The time to disease recurrence is the most important indicator of overall survival, being late recurrence (>2 years from diagnosis) associated with a longer overall survival than early recurrence [19, 22]. Indications for surgery are (1) localized spine mass in patients with neurological symptoms; (2) primary instability or cases with extensive bony involvement where the instability is likely to occur after tumor necrosis resulting from treatment; (3) poor response to initial treatment with chemotherapy or radiotherapy; (4) residual disease; (5) sacral tumors where radical surgical resection is indicated by the aggressive biological behavior and poor prognosis of these lesions. Compared to cases where only decompression or lesionectomy was done, patients who underwent en bloc spondylectomy had a lower recurrence rate [1, 17, 24]. In our case, there was an intradural relapse. One of the most common treatment-related complication is the postlaminectomy kyphosis observed in up to 40% of patients [24]. This is why, whenever possible, enbloc spondylectomy with an anterior column reconstruction is preferable in order to obtain a better oncological control and a better preservation of the spine biomechanics.
